# Lactose intolerance and levothyroxine malabsorption: a review of the literature and report of a series of patients treated with liquid L-T4 without lactose

**DOI:** 10.3389/fendo.2024.1386510

**Published:** 2024-04-11

**Authors:** Silvia Martina Ferrari, Armando Patrizio, Valeria Mazzi, Francesca Ragusa, Chiara Botrini, Giusy Elia, Eugenia Balestri, Emilio Barozzi, Licia Rugani, Fabiana Bracchitta, Giulio Stoppini, Giada Frenzilli, Enke Baldini, Camilla Virili, Salvatore Benvenga, Poupak Fallahi, Alessandro Antonelli

**Affiliations:** ^1^ Department of Clinical and Experimental Medicine, University of Pisa, Pisa, Italy; ^2^ Department of Emergency Medicine, Azienda Ospedaliero-Universitaria Pisana, Pisa, Italy; ^3^ Department of Surgical, Medical and Molecular Pathology and Critical Area, University of Pisa, Pisa, Italy; ^4^ Department of Experimental Medicine, “Sapienza” University of Rome, Rome, Italy; ^5^ Department of Medico-Surgical Sciences and Biotechnologies, “Sapienza” University of Rome, Latina, Italy; ^6^ Department of Clinical and Experimental Medicine, University of Messina, Messina, Italy; ^7^ Adolescent and Women’s Endocrine Health, University of Messina, Messina, Italy; ^8^ Azienda Ospedaliera Universitaria Policlinico “G. Martino”, Messina, Italy; ^9^ Department of Translational Research and New Technologies in Medicine and Surgery, University of Pisa, Pisa, Italy

**Keywords:** liquid L-T4, hypothyroidism, malabsorption, autoimmune thyroiditis, lactose intolerance

## Abstract

In hypothyroid patients needing large doses of levothyroxine (L-T4) (>1.7–2 μg/kg/day) to reach euthyroidism, lactose intolerance (LI) needs to be excluded, owing to the high prevalence in the population. If LI is present, a lactose-free diet decreases the rate of L-T4 malabsorption. However, an increased requirement of L-T4 is described in patients with LI, which can be beneficially treated using lactose-free L-T4 formulation. The lactose-free liquid L-T4 formulation is able to circumvent LI malabsorption leading to the normalization of thyroid-stimulating hormone (TSH) in patients with subclinical hypothyroidism and long-term stable TSH levels.

## 1 Introduction

### 1.1 Lactose intolerance

Intolerance to lactose-containing foods (especially milk derivatives) is frequent and has a prevalence of 7%–20% in Caucasians (people from Northern Europe with the lowest one) and 80%–95% among Native Americans (1). Developmental (linked to prematurity) or congenital lactase deficiency can cause primary lactose malabsorption, even if the genetically determined reduction in lactase activity is more commonly related to racial or ethnic origin (2, 3), while different acquired disorders can induce secondary lactose malabsorption.

The lactase function is active in humans at birth, and it physiologically declines, persisting only in approximately 30% of adults (lactase persistence). Adult hypolactasia (non-persistence of lactase) is common and frequently undiagnosed because its clinical presentation varies and depends on different factors (i.e., the intestinal microflora, the lactose ingested, and the ability to digest it) (4).

The treatment of the primary disorder can restore the lactase activity (5). The typical symptoms, such as flatulence, abdominal pain, diarrhea, bloating, and, especially in adolescents, vomiting, follow the ingestion of lactose, even if a wide variability of symptoms among patients with lactose intolerance (LI) exists. Determinant factors are the rate of gastric emptying, the osmolality and fat content of the food, the rate of intestinal transit, the sensitivity to intestinal distension, and the response of the colon to the carbohydrate load (6). Stools are usually watery, bulky, and frothy. LI can be diagnosed according to the patient’s history, supported by dietary manipulation. Several diagnostic tests are used, ranging from Lactose Breath Test, or changes in serum glucose levels after ingesting standard doses of lactose, to small intestine biopsy. The lactose tolerance test in adult patients has a sensitivity of 75% and a specificity of 96% (7).

A low lactose diet, the administration of enzyme substitute, and calcium and vitamin D supplements can aid in the treatment of lactose malabsorption not associated with a correctable underlying disease (8).

Recently, it has been shown (9) that lactose concentrations <400 mg (overall intake) do not increase breath hydrogen excretion or cause gastrointestinal (GI) symptoms, even though the exact content of lactose in each drug is not always reported in the information leaflets (10, 11).

### 1.2 Lactose and L-T4 malabsorption review of the literature

The diagnosis of LI in adults is not unusual; however, its association with a resistance to the treatment with oral levothyroxine (L-T4) is not frequent (12).

A first investigation by Hays (13) was performed with different experimental L-T4 formulations. The lactose L-T4 preparations that were similar to the commercially available products had a significantly lower absorption rate.

The optimal conditions for the absorption of an L-T4 preparation were evaluated by Wenzel through a double isotope method (14). A tablet similar to a commercial 100 μg L-T4 lactose preparation had a good absorption of 70.6%. Furthermore, the L-T4 was significantly better in the fasting state than with simultaneous food intake, suggesting that simultaneous food intake with L-thyroxine medication should be avoided.

Levothyroxine tablets can undergo degradation and failure to meet potency. A study evaluated how different excipients act on the stability of L-T4 sodium pentahydrate in aqueous slurries and in formulated tablets.

The active L-T4 alone was stable in the solid state for 6 months at 40°C/75% relative humidity (RH) if maintained in open or closed containers and was non-hygroscopic under normal processing conditions (>30% RH).

In aqueous slurries with an excipient, the stability of the active levothyroxine ameliorated once the pH of the slurry was increased from pH 3 to 11. Tablets manufactured with lactose starch, anhydrous, or microcrystalline cellulose failed to meet USP assay requirements at 3 months at 40°C/75% RH. While tablets manufactured with a basic pH modifier and dibasic calcium phosphate, such as sodium carbonate, sodium bicarbonate, or magnesium oxide, met the USP assay requirements at both 3 and 6 months (15).

A 55-year-old woman has been described with clinical signs and symptoms of hypothyroidism, high serum thyroid-stimulating hormone (TSH) (>75 μIU/mL), and low free thyroxine (FT4), due to autoimmune hypothyroidism. In this patient, oral thyroid hormone replacement was started with a dose of 100 μg L-T4/day, and subsequently, the L-T4 dosage was increased step by step up to 900 μg/day in 8 months without significant effects on serum TSH. The patient was found to be affected by undiagnosed oligo-symptomatic LI, and for this reason, a lactose-free L-T4 formulation and dietary lactose restriction were initiated. A normalization of TSH was obtained with 150 μg of lactose-free L-T4. At follow-up, circulating thyroid hormones were determined repeatedly, and the levels remained within the normal range. This study showed that the LI should be considered one of the GI diseases that can cause malabsorption of L-T4 (12).

A further study involved patients with Hashimoto’s thyroiditis (HT) assessing both the prevalence of LI and the effect exerted by a lactose restriction on thyroid function.

Lactose tolerance tests were conducted on all 83 HT patients enrolled who were under an L-T4 therapy.

LI was diagnosed in 75.9% of HT patients. A total of 38 patients with LI (30 euthyroid and eight with subclinical hypothyroidism) and 12 patients without LI initiated a low lactose diet for 8 weeks.

A significant decrease in TSH levels was observed in the euthyroid and subclinical hypothyroid patients with LI (from 2.06 ± 1.02 to 1.51 ± 1.1 IU/mL and from 5.45 ± 0.74 to 2.25 ± 1.88 IU/mL, respectively). However, TSH levels in patients without LI were not significantly modified over 8 weeks. This study showed that LI is highly frequent in HT patients, that LI should be evaluated in hypothyroid patients requiring elevated L-T4 doses, who do not have normal TSH levels and are resistant to L-T4 treatment, and that a low lactose diet leads to diminished levels of TSH (10).

Subsequently, a systematic study assessed the need for T4 in hypothyroid patients with LI. From 2009 to 2012, the replacement T4 dose has been evaluated in 34 hypothyroid patients, due to HT and LI, and non-compliant with a lactose-free diet, in comparison to an age- and sex-comparable group of 68 patients with HT, with no evidence of LI and/or other GI disorders. In controls, target TSH (median TSH, 1.02 mIU/L) was obtained in all patients, with a median T4 dose of 1.31 μg/kg/day. A serum TSH comparable with the one observed in the reference group was obtained in the LI group with a significantly higher dosage, with a median T4 dose of 1.81 μg/kg/day. These findings showed that LI significantly raised the need for oral T4 in hypothyroid patients (4).

The CONTROL Surveillance Project was a patient-based survey performed in hypothyroid patients in treatment. The quantification of the prevalence of factors negatively affecting L-T4 therapy was the primary objective of the study. Among the eligible hypothyroid patients, 925 (92.5%) received levothyroxine monotherapy. About a half of the patients treated with L-T4 (47.0%) had at least one comorbid condition, negatively affecting its absorption: a history of gastric bypass surgery, LI (7.8%), irritable bowel syndrome (9.7%), gastroesophageal reflux disease (33.8%), etc. Other factors adversely affecting L-T4 absorption were the ingestion of medications used to treat comorbid GI conditions, intake of beverages or foods with a high content of fiber, etc. (68.0%), and the use of dietary supplements (51.8%, particularly calcium and iron). Among the 13.4% who controlled their hypothyroid symptoms with difficulty, patients with comorbid GI conditions were significantly more present (7.8 vs. 5.6%, p < 0.01). Patients with GI comorbidities reported two or more doses of L-T4 changes, nearly twice with respect to those without GI disorders (5.0% vs. 3.0%) (16).

Furthermore, we have recently reported five patients with hypothyroidism and LI treated with L-T4 in tablets with lactose, who switched to an oral liquid lactose-free L-T4 formulation at the same dosage and then showed the normalization of serum TSH. Interestingly, three of these patients switched back to the tablets (containing lactose), and this led TSH to rise again. One of these patients was switched from tablet to liquid preparation (with the same dose of L-T4 of 200 μg/day), and TSH decreased from 26.7 to 0.01 μIU/mL. These data suggested that the L-T4 oral liquid formulation could bypass malabsorption in patients with LI (17).

Members of the Associazione Medici Endocrinologi participated in a web-based survey investigating endocrinologists’ use of thyroid hormones in hypothyroid and euthyroid patients in Italy. A total of 797 of 2,028 (39.3%) members completed all the sections of the survey, and most of them (98.7%) indicated L-T4 as the primary treatment of choice for hypothyroidism.

L-T4 in a liquid solution (or soft-gel capsules) is the first choice if it is consumed together with other drugs that interfere with L-T4 absorption (81.8%); in those patients with a history of malabsorption, LI, celiac disease, gastric disorders (such as autoimmune atrophic gastritis) (18), intolerance to common excipients (96.6%), or unexplained poor biochemical control of hypothyroidism (74.4%); or in patients not able to adhere to ingesting L-T4 fasted and/or separated from food/drink (98.9%) (19, 20).

Moreover, a case of a 38-year-old woman was described, who developed severe hypothyroidism after radioactive iodine treatment for Graves’ disease, regardless of a high dose of L-T4 tablet. She was found to have LI and was switched to the powder formulation of L-T4 at the same daily dose, and circulating levels of TSH and thyroid hormones normalized. In this case, the hypothyroidism caused by a malabsorption of L-T4 tablet in a patient with LI was solved by switching to L-T4 powder formulation (21).

In this study, we report our experience in patients with LI and hypothyroidism who were initially treated with “L-T4 in tablet form containing lactose” (L-T4-Tab+Lactose) and then switched to the same dose of “liquid L-T4 (Tirosint, IBSA Farmaceutici Italia Srl, Lodi, Italy) oral solution (lactose-free)” (L-T4-Liq).

## 2 Methods

### 2.1 Patients

In the frame of a wider observational, retrospective evaluation of thyroid hormonal profile in hypothyroid patients who visited our center from January 2013 to August 2021, 18 patients with a new diagnosis of LI were identified. These patients (15 women, three men; age range, 19–68 years; mean age 45 ± 12 years) were affected by hypothyroidism, owing to autoimmune thyroiditis in 12 or thyroidectomized for nodular goiter in six. In all of these patients, hypothyroidism was initially treated with “L-T4 in tablet form containing lactose” (L-T4-Tab+Lactose), with stable TSH value in the normal range in the last 2 years. Because of a new diagnosis of LI, patients were switched from L-T4 in tablet form containing lactose to liquid L-T4. All patients were diagnosed to be affected by LI because of positive Lactose Breath Test and were taking a low lactose diet. Furthermore, all the patients were investigated for other GI diseases, and, to avoid bias in the evaluation of T4 malabsorption, only patients with negative results were included in the data analysis: a) clinically by the exclusion of anemia associated with cobalamin deficiency or iron deficiency and b) measuring anti-tissue transglutaminase IgG and IgA antibodies, gastrin, antiparietal cell antibodies, antiendomysial IgA and IgG antibodies, *Helicobacter pylori* (HP) antigen in the stool (4). Other conditions related to L-T4 malabsorption were not present, such as a) concurrent therapy with amiodarone, beta-blockers, orlistat, lithium, raloxifen, interferons, cholestyramine, antiacids, or proton-pump inhibitors (PPIs) (22, 23) and b) preceding bariatric or gastric or intestinal surgery. Moreover, we excluded pseudo-malabsorption owing to poor compliance in each patient.

The investigator sought for written informed consent to the data analysis from all subjects. The study was conducted in accordance with the Declaration of Helsinki, and the protocol was approved by the local ethical committee (Comitato Etico di Area Vasta Nord Ovest per lo studio ID n. 16596/2020).

### 2.2 Study design

The data used for this study were routinely collected from patients during their visits to our clinical site as recorded in the medical records. Data were abstracted from the medical records and entered into a database. Extracted data were anonymized with unique identifiers. All the patients included in the data analysis were switched to liquid L-T4 (Tirosint, IBSA Farmaceutici Italia Srl, Lodi, Italy, containing as excipients: 96% ethanol; glycerol 85%), from the L-T4 tablets (from different pharmaceutical companies; T-LT4 group), with the same dosage (L-T4, 1.52 ± 0.81 μg/kg/day), administered 30 min before breakfast. Circulating TSH, FT4, and free triiodothyronine (FT3) were recorded before the switch, and again after 2, 12, and 24, months from the switch. The patients were not treated with any other interfering drug.

L-T4 was then supplied again at the same dosage in tablets in six patients who wished to switch back to the tablet form. The reasons why six patients wished to switch back to the tablet were the following: a) five patients found it easier to take tablets than the liquid vials, and b) one patient did not like the liquid taste. Circulating levels of FT4 (normal range, 0.7–1.7 ng/dL), FT3 (normal range, 2.7–4.7 pg/mL), and TSH (normal range, 0.4–4 μIU/mL) were tested. At baseline and after the switch, the level of each hormone was obtained from the mean of the two samples collected before the L-T4 dose.

### 2.3 Data analysis

Values are given as mean ± SD for normally distributed variables, alternatively as median and [interquartile range]. One-way analysis of variance (ANOVA) was performed to compare mean group values (for normally distributed variables, such as body mass index (BMI) and age); *post-hoc* comparisons were done by Bonferroni–Dunn test. Chi-square test was done to compare the proportions. Student’s two-tailed t-test was used to compare two groups.

The correlation among changes in TSH (after the switch to liquid L-T4—baseline with tablet L-T4) vs. changes in FT4 or FT3 was evaluated by simple regression.

## 3 Results

The evaluation of thyroid hormones before the switch revealed that seven patients had subclinical hypothyroidism [TSH level in the hypothyroid range (> 3.5 μIU/mL), with normal FT3 and FT4], while the others had normal values of TSH, FT3, and FT4.

All 18 patients were evaluated after approximately 2 months (first control: after 54 ± 12 days from the initial switch). Further evaluations in the 12 patients who continued liquid L-T4 were made after approximately 12 months and 24 months. In the patients who switched back to tablet-LT4, thyroid hormones re-evaluation was made after 2 months. Body weight of patients was not significantly changed over time [BMI, base 24.2 ± 2.7 kg/m^2^; first control, 24.7 ± 2.9 kg/m^2^; second control, 24.5 ± 2.7 kg/m^2^].

**Figure 1:**
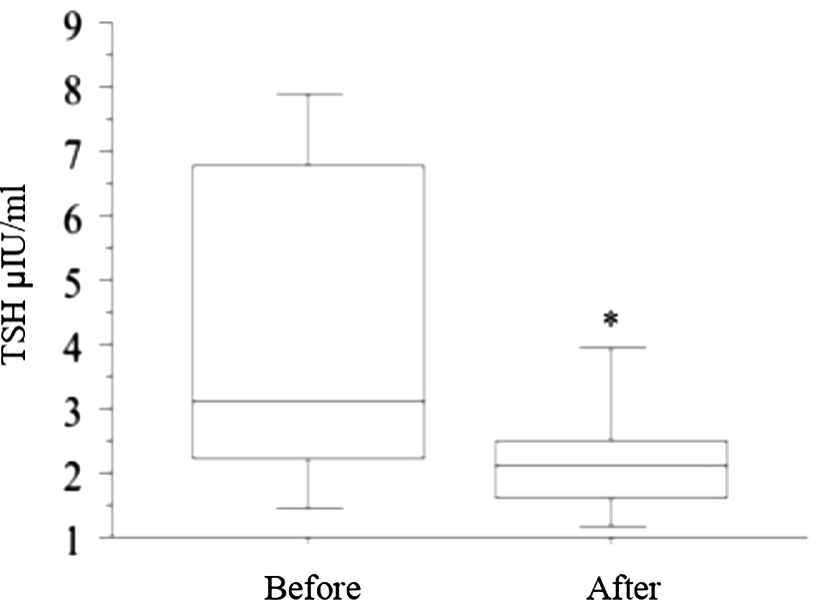
The switch from tablet L-T4 to liquid L-T4 (reported in the figure with “Before” and “After”, respectively), was followed by a significant decline in TSH values with respect to the basal value (* p = 0.007, t-test) after 2 months.

The switch to liquid L-T4 was followed by a significant decline of TSH values respect to the basal value (p = 0.007, t-test) (**Figure 1**), while FT4 (1.21 ± 0.26 vs. 1.25 ± 0.28, ng/dL) and FT3 (2.89 ± 0.81 vs. 2.91 ± 0.92, pg/mL) levels were not significantly changed.

Patients affected by autoimmune thyroiditis showed a decline in TSH values (with respect to the basal value) after the switch to liquid L-T4, similar to that observed in thyroidectomized ones (data not shown), without any significant difference between the two groups.

**Figure 2:**
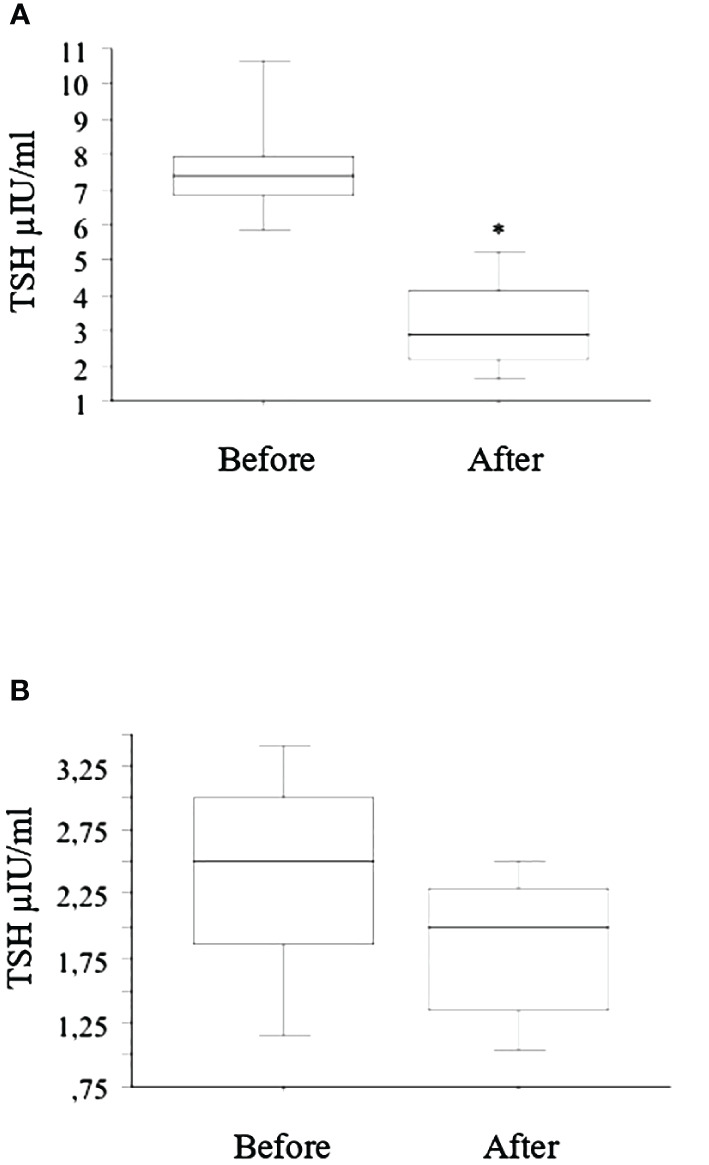
After the switch from tablet to liquid L-T4 (reported in the figure with “Before” and “After”, respectively), the seven patients with LI and subclinical hypothyroidism had a significant (* p < 0.01, ANOVA) reduction in TSH after 2 months **(A)**, while in patients with basal normal TSH value, a slight and not significant decrease was observed **(B)**.

The seven patients with LI and subclinical hypothyroidism had a significant reduction in TSH (**Figure 2A**), while in patients with basal normal TSH value, a slight and not significant decrease was observed (**Figure 2B**).

A simple regression (r = 0.621, p = 0.01) showed a negative correlation between the decrease in TSH (following the switch to liquid L-T4—baseline with tablet L-T4) vs. the increase in FT4 (following the switch to liquid L-T4—baseline with tablet L-T4). Moreover, no significant association was reported between changes in FT3 and changes in TSH (following the switch to liquid L-T4—baseline with tablet L-T4).

**Figure 3:**
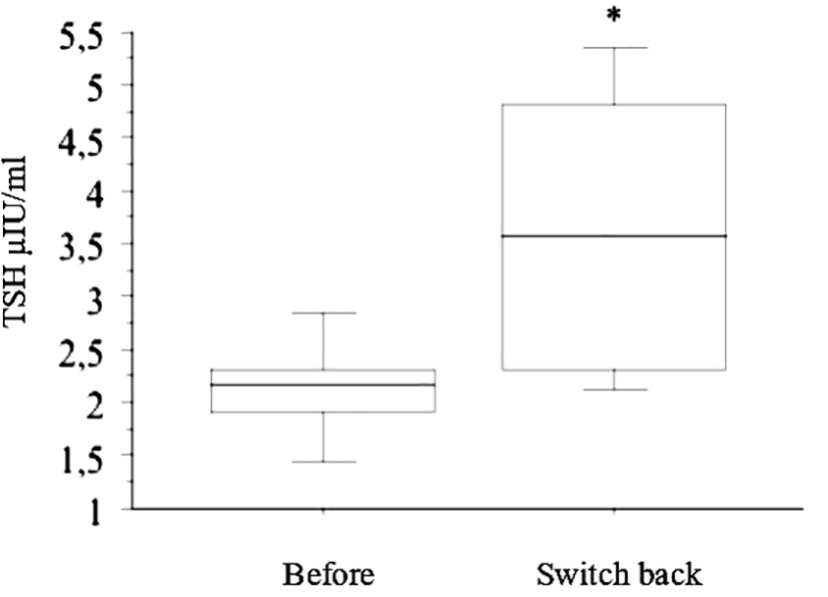
In the six patients who switched back from liquid L-T4 to tablets (reported in the figure with “Before” and “Switch back”, respectively), TSH increased significantly again (* p < 0.05, by ANOVA).

TSH increased significantly again in the six patients who switched back to tablets (**Figure 3**). All of them were treated again with liquid-L-T4 with normalization of TSH.

**Figure 4:**
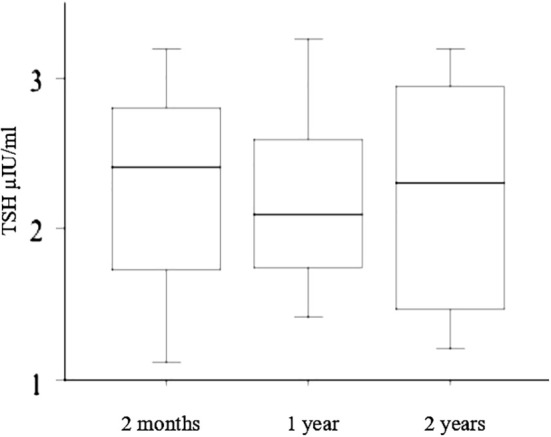
Since the liquid L-T4 formulation resulted in a better control of TSH levels, all patients (included those who had been temporarily switched back to tablets) were finally treated with the liquid L-T4, and TSH, FT3, FT4 were evaluated again 2 months after the initial evaluation, and after 12 months and 24 months (reported in the figure with “2 months”, “1 year”, and “2 years”, respectively) resulting in the normal range in all subjects.

As the liquid L-T4 formulation showed a better control of TSH levels, all patients (included those who had been temporarily switched back to tablets) were lastly treated with the liquid L-T4, and TSH, FT3, FT4 were tested again 2 months after the initial evaluation, and after 12 months and 24 months, and were in the normal range in all subjects (**Figure 4**).

## 4 Discussion

The need to treat patients with hypothyroidism with high L-T4 doses is often the very first sign of one of the disorders associated with malabsorption syndrome (24–29). Considering the patients needing elevated doses of L-T4 (>1.7–2 μg/kg/day) to reach euthyroidism (23, 30), first, a pseudo-malabsorption should be excluded due to poor compliance.

Moreover, other factors linked to L-T4 malabsorption, such as medications or bowel and gastric diseases, should be investigated (26). LI should be investigated in the differential diagnosis of GI diseases causing L-T4 malabsorption (26). In Caucasian adult patients, the prevalence of LI is 7%–20%, but it can be frequently ignored (26).

The pathogenic mechanisms that lead to an increased T4 need in patients with LI are still unknown. LI can occur when, owing to a lactase deficiency in the small intestinal brush border, an elevated quantity of lactose is not absorbed and remains undigested, attracting water into the intestinal lumen and leading to bacterial fermentation.

Many reasons can explain how the absorption of oral T4 may be affected in patients with LI while on a diet containing lactose. First, similarly to what was observed with various drugs, oral T4 can be adsorbed and trapped by the modified intestinal content (31). Second, a faster intestinal transit reduces the exposure time for T4 interaction with intestinal mucosa, decreasing the bioavailability of T4 (32). The different microbiota of patients with LI can alter the villous architecture, which is of primary importance for the correct absorption of nutrients and drugs (4, 8, 33–35). Recently, the important role of an altered gut microbiota composition in the pathogenesis and progression of several autoimmune disorders has been shown. In autoimmune thyroid disorders, and among them in autoimmune thyroiditis, a predisposing genetic background and possible environmental triggers can lead to the loss of self-tolerance, involving cellular and humoral responses of the immune system. Different autoimmune disorders have a pathogenetic link with dysbiosis, and as intestinal dysbiosis occurs, the epithelial barrier fails to function and intestinal and systemic disorders can appear. Hypothyroidism is associated with bacterial overgrowth in small intestinal or with changes in the composition of microbiota (36, 37).

A lactose-free diet decreases the rate of L-T4 malabsorption. The symptoms of LI improve in 2–3 weeks upon lactose restriction (38), while TSH levels are affected after approximately 4–6 weeks (10). However, the L-T4 requirement in patients with LI, treated with L-T4 tablets containing lactose, remains high (10).

In fact, a lactose-restricted diet includes limiting the consumption of milk derivatives and may be nutritionally not advantageous, as a reduced intake of calcium and phosphorus can cause bone demineralization. Furthermore, a lactose-free diet is difficult to achieve owing to the sneaky ingestion of this disaccharide, which is present as a preservative in many foods.

Furthermore, L-T4 lactose preparation, which was prepared similarly to commercially available products, might have a significantly lower absorption in LI patients. Lactose is often present as a supplementary ingredient in many commercially available drugs (11), as in L-T4 preparations. In susceptible individuals, drugs containing lactose can cause LI symptoms (11), and lactose in L-T4 preparations could impair thyroxine absorption (12).

Lactose is contained obviously in milk and other foods that are normally ingested with breakfast. Many studies have reported that the L-T4 absorption, using tablet, is impaired if the drug is taken together with breakfast or foods. The impairment could be theoretically higher in patients with LI; however, no study has precisely evaluated the rate of L-T4 absorption using tablet with lactose in this specific condition. Moreover, as underlined by other studies (12), undiagnosed oligo-symptomatic lactose intolerances may account for other cases of L-T4 malabsorption.

Regarding L-T4 therapy, the most recent advancement is the new oral liquid preparation (39), able to avoid different malabsorption conditions (22, 40–45).

A recent paper evaluated five patients with autoimmune gastritis in therapy with L-T4 tablets, with high circulating TSH levels (in the hypothyroid range). Circulating TSH levels normalized once all the patients who received L-T4 in tablet form were switched to an oral L-T4 liquid formulation with the same dosage. Four patients were switched back to L-T4 tablets, maintaining the dosage, and TSH reached again the hypothyroid range. These results suggest that the L-T4 oral liquid formulation could circumvent the pH alteration caused by atrophic gastritis (42).

Furthermore, we have reported previously five patients with hypothyroidism and LI during the therapy with L-T4 in lactose-containing tablets, in whom the switch to an oral liquid lactose-free L-T4 formulation with the same dosage led circulating TSH to normalize. Of note, in three of these patients, the switch from the liquid formulation to the lactose-containing tablets caused TSH to worsen again (17).

The results reported in the present study agree with the above-mentioned paper and reinforces the concept that L-T4 liquid preparation can circumvent malabsorption in patients with LI. Interestingly, the L-T4 liquid preparation is associated with a larger decrease in TSH in patients with LI and subclinical hypothyroidism with respect to patients who were euthyroid at the moment of the switch, similarly to what was observed in other studies (10).

Furthermore, the liquid treatment was associated with stable TSH levels during the 2 years of follow-up in the study. This suggests that the liquid L-T4 can abolish the need for frequent L-T4 dose adjustments, which has been recently described in patients with GI comorbidities in other studies (14). We have found that FT3- and FT4-circulating levels were not significantly different between the two groups during the different controls. This is absolutely in line with the results of many other studies and of our studies that evaluated the efficacy of different formulations of L-T4. In fact, TSH levels are more sensitive than thyroid hormone levels to evaluate the hormonal homeostasis in patients with hypothyroidism (46).

Moreover, in the present study, L-T4 was then supplied again at the same dosage in tablets in six patients (33%) who wished to switch back to the tablet form, for different reasons: a) five patients found it easier to take tablets than the liquid vials, and b) one patient did not like the liquid taste. In these six patients, TSH increased significantly again (p < 0.05, by ANOVA).

Different reasons can explain the increased effectiveness of liquid L-T4. First of all, the liquid-L-T4 that we used is lactose-free. Second, pharmacokinetic studies have shown a more rapid L-T4 absorption with the liquid formulation, with respect to the tablets, and this may counteract the faster intestinal transit in LI. Third, the liquid formulation absorption might initiate in the oral mucosa, partially overcoming the intestinal absorption (47, 48). However, other studies are needed to evaluate the exact mechanism.

A recent double-blind, randomized study has shown that the liquid preparation can overcome the L-T4 absorption defect due to the breakfast. This might be due, at least partially, to an increased L-T4 absorption in patients with undiagnosed oligo-symptomatic lactose intolerances (41).

## 5 Conclusion

Refractory hypothyroidism and the need to increase the “normal dose” of L-T4 has led to the development of novel L-T4 formulations, which have allowed a significant reduction in TSH variability in hypothyroidism, with respect to tablets (49).

Many studies have shown that LI is associated with impaired L-T4 absorption in patients affected with hypothyroidism. In patients needing elevated doses of L-T4 (>1.7–2 μg/kg/day) to reach euthyroidism, first, pseudo-malabsorption should be excluded, as well as other factors related to L-T4 malabsorption (as medications, or bowel and gastric diseases). However, owing to the high prevalence in the population, LI needs to be excluded, too. If LI is present, a lactose-free diet leads to a decline in the rate of L-T4 malabsorption.

However, an increased requirement of L-T4 is described in patients with LI, which can be beneficially treated using lactose-free L-T4 formulation. The lactose-free liquid L-T4 formulation is able to circumvent LI malabsorption leading to the normalization of TSH in patients with subclinical hypothyroidism and long-term stable TSH levels (46, 50).

Further investigations will be needed to evaluate the liquid L-T4 formulations in larger numbers of patients with hypothyroidism and LI, also in patients with TSH values in the reference range under L-T4 tablet therapy.

## Data availability statement

The raw data supporting the conclusions of this article will be made available by the authors, without undue reservation.

## Ethics statement

The studies involving humans were approved by Comitato Etico di Area Vasta Nord Ovest. The studies were conducted in accordance with the local legislation and institutional requirements. The participants provided their written informed consent to participate in this study.

## Author contributions

SMF: Data curation, Formal analysis, Writing – original draft, Writing – review & editing. AP: Data curation, Formal analysis, Writing – original draft, Writing – review & editing. VM: Writing – review & editing. FR: Writing – review & editing. CB: Writing – review & editing. GE: Writing – review & editing. EuB: Writing – review & editing. EmB: Writing – review & editing. LR: Writing – review & editing. FB: Writing – review & editing. GS: Writing – review & editing. GF: Writing – review & editing. EnB: Writing – review & editing. CV: Writing – review & editing. SB: Writing – review & editing. PF: Data curation, Formal analysis, Writing – original draft, Writing – review & editing. AA: Data curation, Formal analysis, Writing – original draft, Writing – review & editing.
